# PolyQ 2.0: an improved version of PolyQ, a database of human polyglutamine proteins

**DOI:** 10.1093/database/baw050

**Published:** 2016-05-17

**Authors:** Chen Li, Jeremy Nagel, Steve Androulakis, Christopher J. Lupton, Jiangning Song, Ashley M. Buckle

doi:10.1093/database/baw021

The authors regret the omission of co-author Christopher J. Lupton from the original publication of the paper ‘PolyQ 2.0: an improved version of PolyQ, a database of human polyglutamine proteins’. The authorship list should have read as follows:

Chen Li^1^, Jeremy Nagel^1^, Steve Androulakis^2^, Christopher J. Lupton^1^, Jiangning Song^1,3,^*, and Ashley M. Buckle^1,^*

^1^Biomedicine Discovery Institute and Department of Biochemistry and Molecular Biology, ^2^Monash Bioinformatics Platform, Monash University, Melbourne, Vic. 3800, Australia, and ^3^National Engineering Laboratory of Industrial Enzymes and Key Laboratory of Systems Microbial Biotechnology, Tianjin Institute of Industrial Biotechnology, Chinese Academy of Sciences, Tianjin 300308, China

